# A surgical case of pulmonary adenocarcinoma in the right upper lobe associated with a systemic artery‐to‐pulmonary artery fistula

**DOI:** 10.1111/1759-7714.14985

**Published:** 2023-06-12

**Authors:** Yuta Ishida, Takuro Yukawa, Yasunari Nagasaki, Daisuke Minami, Hiroyasu Fujiwara, Yasumasa Monobe, Takuya Fukazawa, Tomoki Yamatsuji

**Affiliations:** ^1^ Department of General Surgery Kawasaki Medical School Okayama Japan; ^2^ Department of General Internal Medicine 4, Kawasaki Medical School Okayama Japan; ^3^ Department of Diagnostic and Therapeutic Radiology Kawasaki Medical School Okayama Japan; ^4^ Department of Pathology 1 Kawasaki Medical School Okayama Japan

**Keywords:** lung cancer, selective arterial embolization, systemic artery‐to‐pulmonary artery fistulas

## Abstract

A 52‐year‐old female never‐smoker with an abnormal shadow in the right lung detected on radiography was referred to our institution. Contrast‐enhanced computed tomography revealed an irregular nodule in the upper lobe of the right lung, suggestive of a pulmonary vascular abnormality. Angiography revealed a direct communication between the right internal mammary artery (IMA) and the right upper lobe pulmonary artery branches, with dilated and tortuous vascular proliferation. As multiple branch arteries were seen flowing into the upper lobe from the IMA, transcatheter selective embolization of these vessels and right upper lobectomy by video‐assisted thoracoscopic surgery were performed. Contrary to the clinical diagnosis, the pathological finding was a pulmonary adenocarcinoma of the right upper lobe. Additional lymph node dissection was performed later. We report an extremely rare and unprecedented case of pulmonary adenocarcinoma fed by the right IMA, with a literature review.

## Graphical abstract

In this report, we present an extremely rare and unprecedented case of pulmonary adenocarcinoma receiving its nutritional supply from the right internal mammary artery (IMA). The patient was diagnosed with a systemic arterio‐pulmonary fistula (SAPAF) and underwent a right upper lobectomy due to the lesion's growth over time. However, pathological examination revealed that the lesion was a pulmonary adenocarcinoma associated with SAPAF.

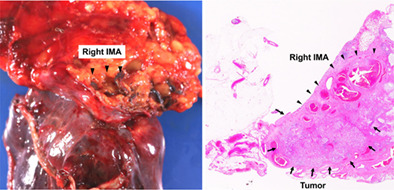

## INTRODUCTION

Diseases, such as lung cancer, tuberculosis, and pulmonary arteriovenous malformations (PAVMs) present as nodular shadows on chest radiography. Systemic arterio‐pulmonary artery fistulas (SAPAFs) and systemic arterio‐pulmonary vessel fistulas, which are circulatory shunts from systemic arteries to pulmonary vessels, are rare diseases that appear as nodular shadows on chest radiography.^1^, ^2^ Bronchoscopy is performed to evaluate for malignancies, pulmonary mycobacteriosis, or other diseases. However, bronchoscopy is contraindicated for vascular malformations due to an increased risk of bleeding.^3^ Here, we report a rare surgical case of pulmonary adenocarcinoma, preoperatively diagnosed as SAPAF. This case highlights that malignant tumors can further complicate pulmonary vascular abnormalities.

## CASE REPORT

**FIGURE 1:**
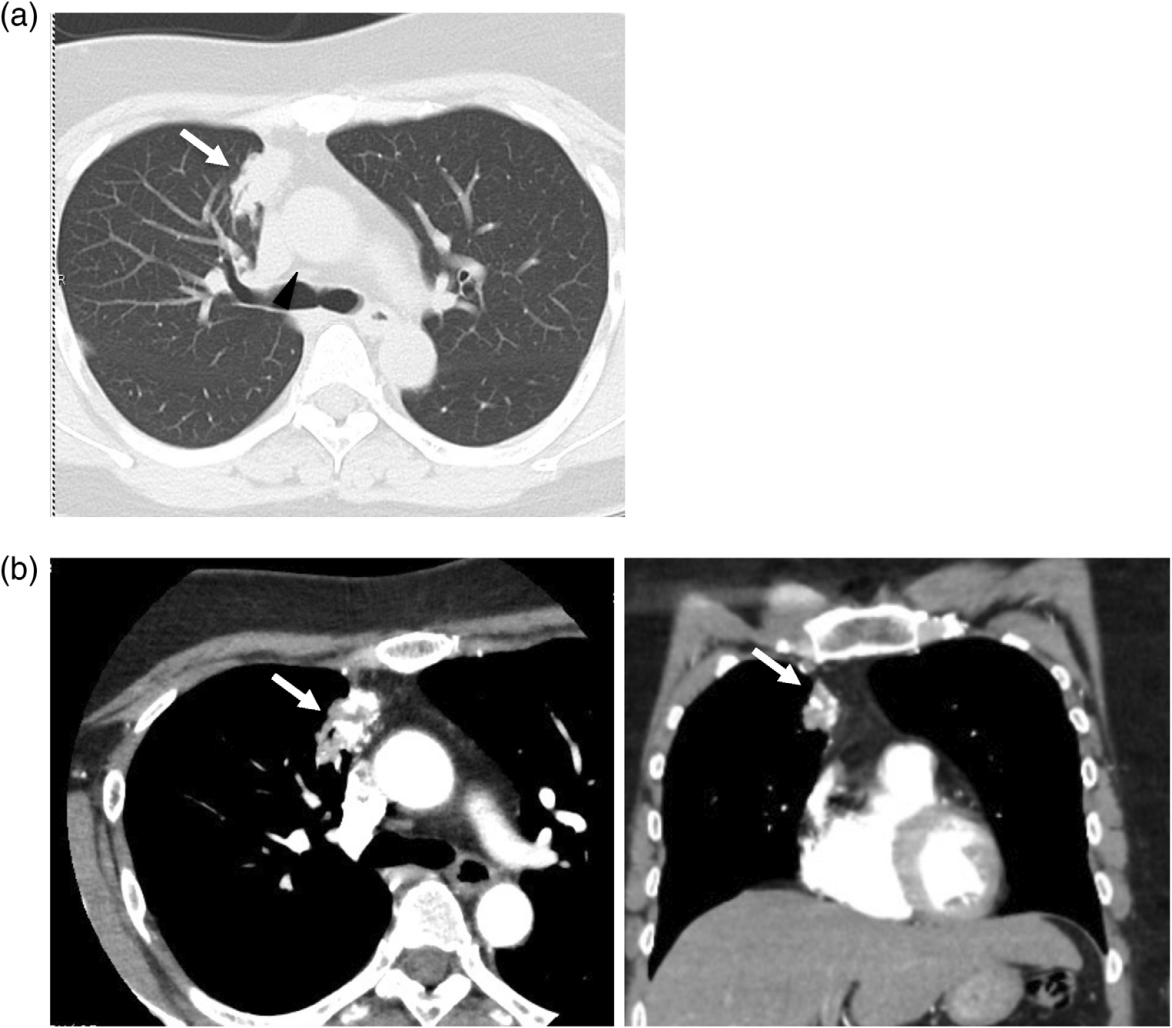
(a) Computed tomography (CT) showing a 31‐mm irregular nodule in the anterior segment (S3) of the upper lobe of the right lung (white arrow). (b) Contrast‐enhanced CT showed a tubular structure (white arrow).

**FIGURE 2:**
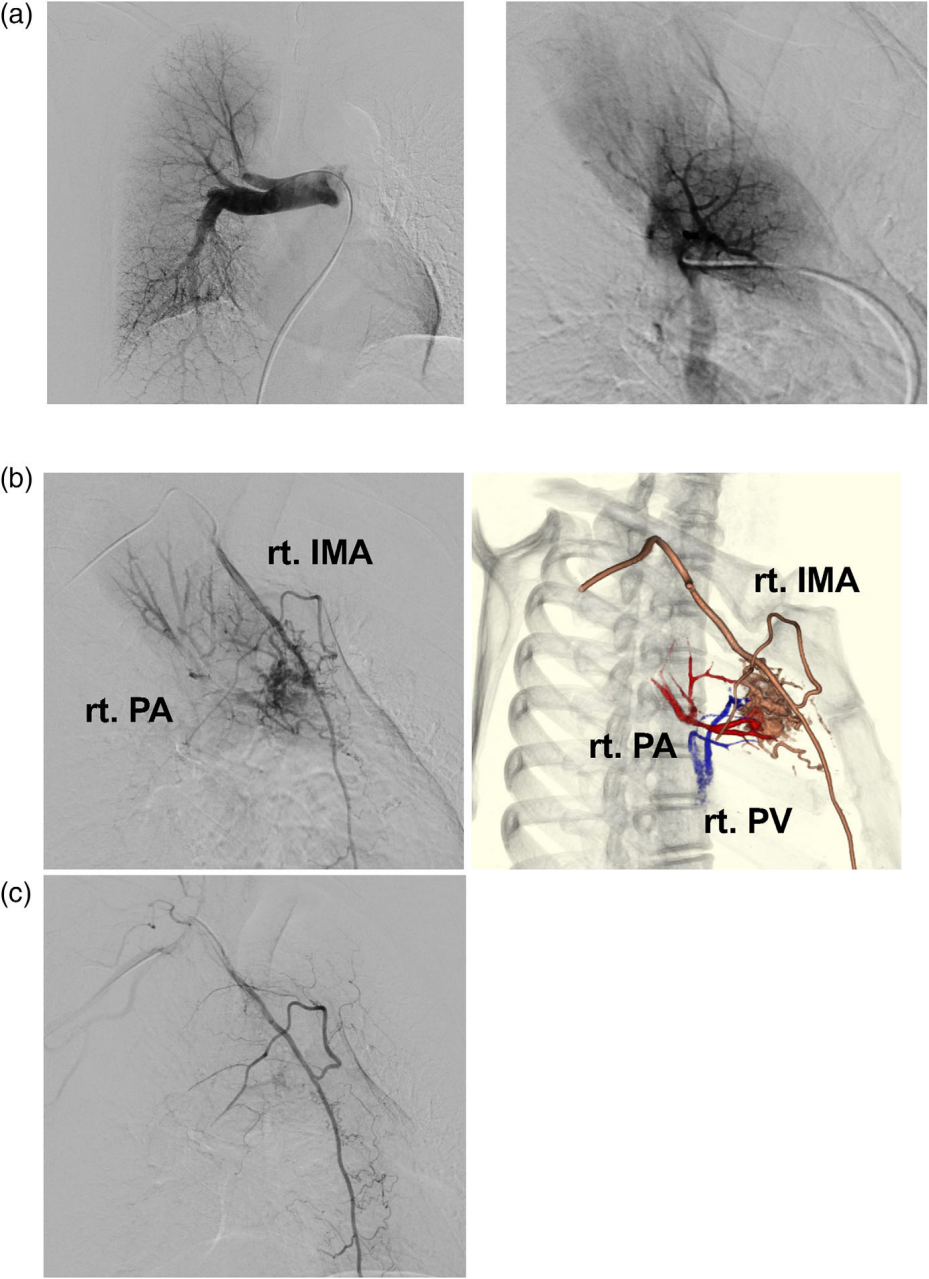
(a) Right pulmonary arteriography showed pulmonary arteries distributed around the lesion, but no arteriovenous shunt was observed. (b) Selective right internal mammary arteriography showed markedly dilated tortuous vascular growth and communication with the right pulmonary artery. IMA, internal mammary artery, PA, pulmonary artery, PV, pulmonary vein. (c) After embolization of the branch of the internal mammary and distal feeders to the fistula, only minimal filling of the network of aberrant blood vessels was identified. No filling of the pulmonary artery was seen.

**FIGURE 3:**
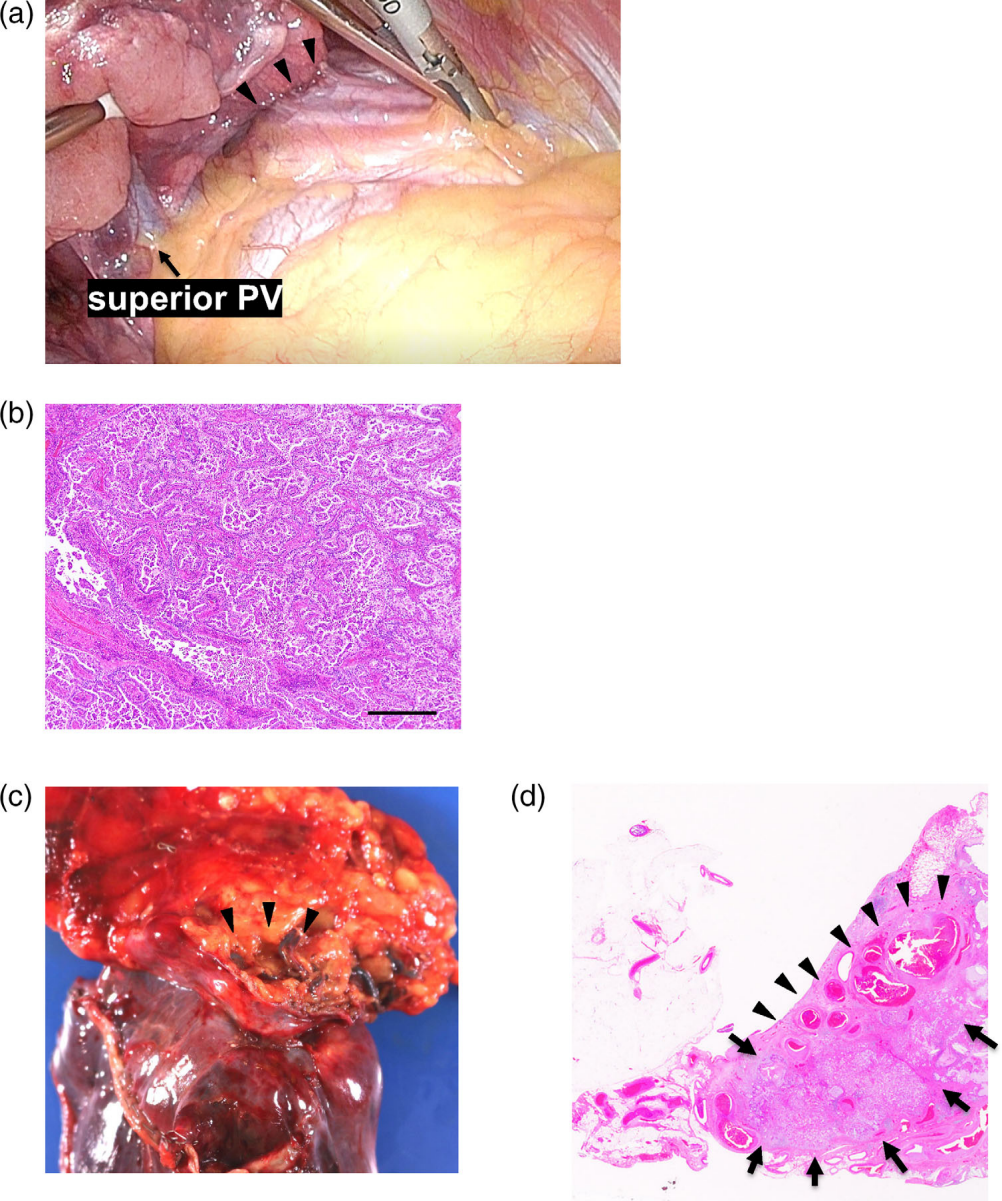
(a) Thoracoscopic findings showed that the right upper lobe was adherent to the anterior mediastinum. PV, pulmonary vein. (b) Microscopic findings of papillary adenocarcinoma. Scale bar, 200 μm. (c) Macroscopic findings in the right upper lobe and anterior mediastinal adipose tissue. A right medial mammary artery fragment can be seen (arrowhead). (d) Pulmonary adenocarcinoma (arrow) and right internal mammary artery inflow (arrowhead) were observed in a loupe view using hematoxylin–eosin staining.

A 52‐year‐old female never‐smoker, with an abnormal shadow in the right lung detected on radiography, was referred to our institution. Computed tomography (CT) revealed an irregular nodule in the upper lobe of the right lung (Figure 1a). Contrast‐enhanced CT revealed a tortuous vascular component inside the nodule (Figure 1b), with high signal intensity on T2‐weighted magnetic resonance imaging (MRI) and gradual contrast on dynamic MRI (Figure S1a,b), suggestive of pulmonary vascular malformation. Though catheter angiography was performed under the suspicion of a pulmonary arteriovenous fistula, no communication between the pulmonary artery and vein was detected (Figure 2a). Selective right internal mammary arteriography revealed communication with the right pulmonary artery (Figure 2b). Because the lesion gradually increased in size, a video‐assisted thoracic surgery (VATS) right upper lobectomy was performed for definitive diagnosis and treatment. Selective embolization of the branch arteries arising from the internal mammary artery (IMA) was preoperatively performed by a radiologist (Figure 2c). Thoracoscopic findings revealed an intrathoracic adhesion between the right upper lobe and anterior mediastinum (Figure 3a) and the mediastinal fatty tissue was resected with the lung. Positron emission tomography (PET) was not performed due to the low malignant risk of the lesion. However, pathological examination revealed a pulmonary adenocarcinoma (invasive adenocarcinoma with micropapillary carcinoma, Figure 3b) associated with a SAPAF. Intraoperatively, no effusion or dissemination was observed. The tumor measured 32 × 22 mm, and invaded the visceral pleura. It was pathologically diagnosed as pT4 due to invasion of the mediastinal fat tissue. The right IMA was confirmed to be the feeding artery of the tumor (Figure 3c, d).

At a later date, additional hilar and upper mediastinal lymph node dissection was performed. The pathological stage was pT4N0M0, stage IIIA. No *EGFR* mutations, *ALK* fusion genes, or BRAF^V600E^ were detected, and PD‐L1 expression was low. Her postoperative course was uneventful, and she was discharged on postoperative day 11. She received four cycles of adjuvant chemotherapy with cisplatin and pemetrexed. No tumor recurrence was observed 11 months after the treatment.

## DISCUSSION

SAPAF occurs due to pulmonary sequestration,^4^ congenital heart disease,^5^ tuberculosis,^6^ Takayasu's arteritis,^7^ trauma,^8^ and thoracotomy.^9^ The most commonly reported systemic arteries involved are the internal mammalian, intercostal, and subclavian arteries.^10^ Abnormal chest shadows, hemoptysis, heart murmurs, and anginal symptoms after coronary artery bypass surgery are known clues for diagnosing SAPAF.^11^, ^12^, ^13^ Unfortunately, these disease‐specific symptoms were absent in the present case.

Catheter angiography of the circulatory system is vital in diagnosing a pulmonary vascular malformation. In our case, pulmonary arteriography ruled out PAVM because no circulatory shunt was detected between the pulmonary artery and pulmonary vein. Instead, communication between the right IMA and right pulmonary artery, through the pleura, was observed. Lung resection or ligation of the inflow vessels is performed for SAPAF treatment.^1^, ^11^ However, vascular embolization^14^ has been increasingly reported. In this case, since the lesion had grown over time, a lobectomy was performed to make a definitive diagnosis. Enucleation or partial resection was not performed to avoid intraoperative hemorrhage.

According to a report of 80 cases of intrapulmonary vascular involvement of the internal thoracic artery,^15^ 85% of the cases were acquired. In addition to inflammatory disease, chest trauma, and thoracic surgery, malignant lymphoma was reported by Dunn et al.^16^ as an underlying disorder. They reported angiographic findings of blood shunting from the left mammalian artery into the left pulmonary artery, via an intrapulmonary Hodgkin's lymphoma infiltrating the anterior mediastinum. However, it was unclear whether the lymphoma drew blood flow from the systemic artery to the pulmonary artery, or whether pre‐existing vascular abnormalities promoted tumorigenesis.

In our case, pathological examination confirmed that the IMA passed through the resected mediastinal tissue into the lung parenchyma, suggesting that the right IMA nourished the tumor and the involvement of the systemic‐pulmonary fistula.

As the patient had an unremarkable medical history, there was no evidence that proved the complete absence of other congenital factors. It was unknown whether the lung cancer was the real cause of SAPAF development. It also remains unclear whether the vascular involvement abnormally promoted tumor formation, as in the case of Hodgkin's lymphoma described above.

This study reported a case of pulmonary adenocarcinoma associated with SAPAF. A lung cancer fed by a systemic artery has not previously been reported. Clinicians should be aware that pulmonary vascular abnormalities might be further complicated. Even if pulmonary vascular abnormalities are suspected preoperatively, intraoperative pathology might be advisable if an elastic hard mass is palpable in the resected lung.

## AUTHOR CONTRIBUTIONS

Yuta Ishida and Takuya Fukazawa wrote the manuscript with input from all authors. All authors approved the final version of the manuscript before submission.

## CONFLICT OF INTEREST STATEMENT

The authors report no competing interest.

## Supporting information


Figure S1.
