# Supplementary figures and images for: A surgical case of pulmonary adenocarcinoma in the right upper lobe associated with a systemic artery‐to‐pulmonary artery fistula
Source: Thorac Cancer. 2023 Jun 12;14(21):2085–9. doi: 10.1111/1759-7714.14985 (PMC10363778; doi:10.1111/1759-7714.14985)

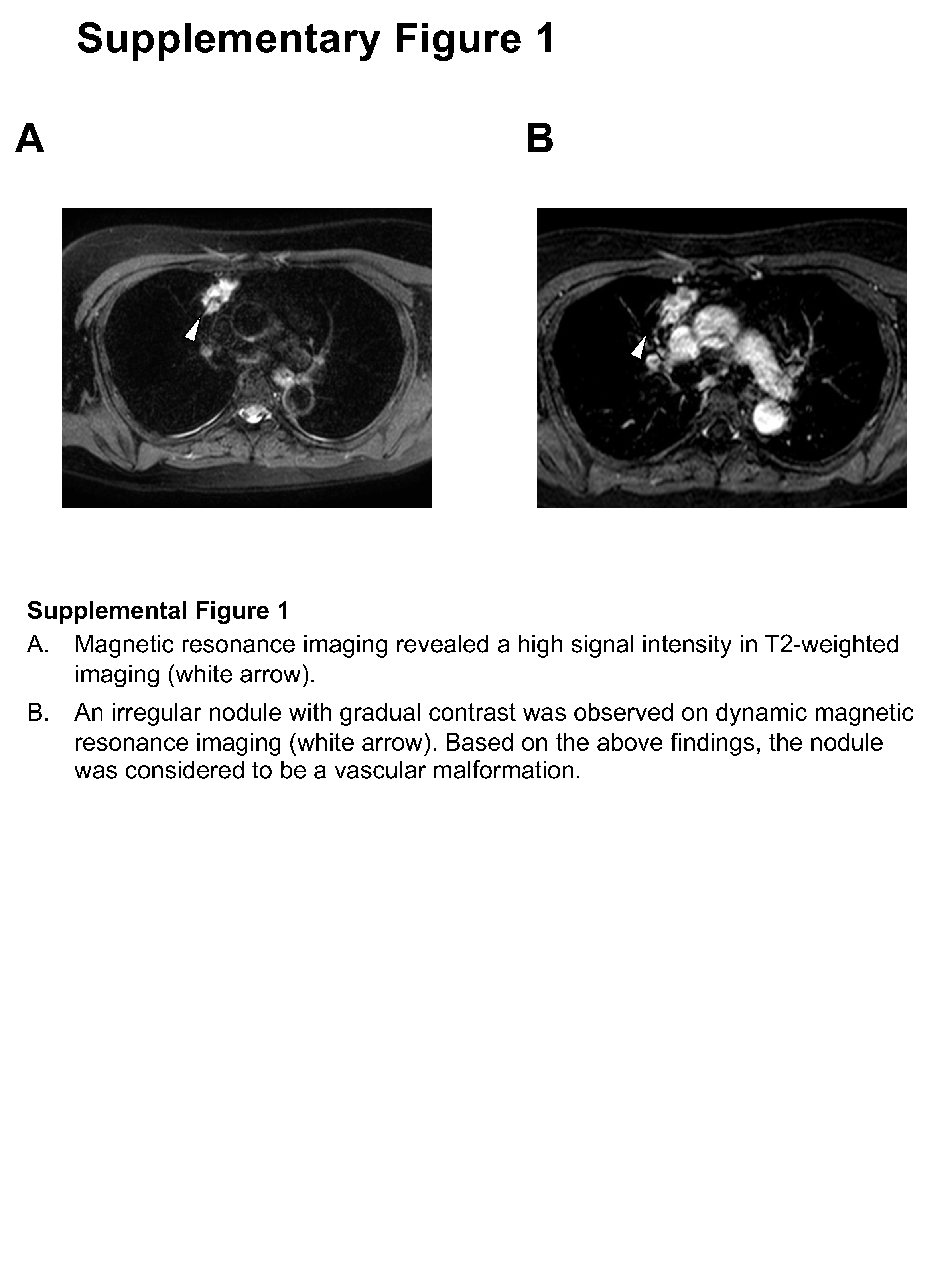

Supplement: Supplementary file 1 — Figure S1. [file TCA-14-2085-s001.tif]
